# Supplementary material for: Se-O Bond Is Unique to High Se Enriched Sweet Potato Stem Protein with Better Antioxidant Ability
Source: Foods. 2021 Dec 9;10(12):3064. doi: 10.3390/foods10123064 (PMC8701230; doi:10.3390/foods10123064)
Supplement: Supplementary file 1 [file foods-10-03064-s001.zip › foods-1450811-Supplementary.pdf]

Supplementary Data (Foods)

Se-O bond is unique to high Se enriched sweet potato stem protein with better antioxidant ability

Qi Gao, Jia-Le Wu, Lan-Ping Jiang, Su-Qi Sun, Xue-Jun Gu, Mei Tie, Masaru Tanokura, You-Lin Xue

Contents:

Supplementary Figure S1. SEM-EDS of the SSP samples: (A) SSP, (B) low-Se SSP and (C) high-Se SSP.

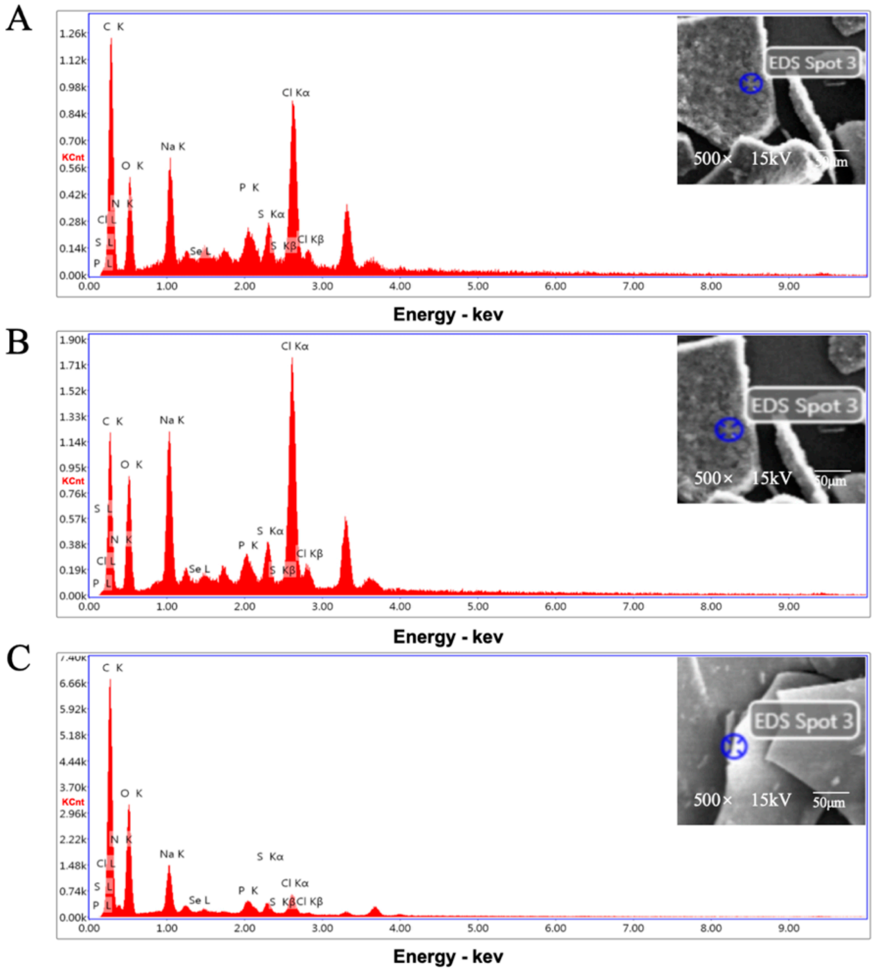

Figure S1. Gao *et al.*
